# Spatial analysis of fast-food accessibility and obesity among adults in Malaysia nationwide

**DOI:** 10.1371/journal.pone.0335725

**Published:** 2025-12-02

**Authors:** Kimberly Yuin Y’ng Wong, Foong-Ming Moy, Sanjay Rampal

**Affiliations:** 1 Department of Social and Preventive Medicine, Faculty of Medicine, University of Malaya, Kuala Lumpur, Malaysia; 2 Centre for Nutrition Epidemiology Research, Institute for Public Health, National Institutes of Health, Ministry of Health, Malaysia; Georgetown University Medical Center, UNITED STATES OF AMERICA

## Abstract

With the rising prevalence of obesity in Malaysia, addressing the obesogenic environment is increasingly urgent. While fast-food environments were linked to obesity and poorer diet quality in Western populations, evidence on this association in Malaysia remains limited. This study evaluated the association between accessibility to fast-food restaurants (FFR) and Body Mass Index (BMI) among adults aged 18–59 years from the National Health and Morbidity Survey (NHMS) 2015. After exclusions due to data requirements, 14,584 respondents from the initial 19,935 were included. FFR locations were obtained from websites of major franchises. Fast-food (FF) proximity was distance to the nearest FFR from each respondent’s residence. FF spatial access was the sum of inverted distances to all FFR within an 8 km Euclidean distance and subsequently categorized into none, low, moderate, and high. Multilevel linear regression adjusted the associations of BMI for socio-demographics, district population density, and district median income. Among the respondents studied (51.5% female), mean BMI was 26 kg/m^2^ and median FF proximity was 2.6 km, where 77.3% had spatial access to at least one FF restaurant within 8 km from their residence. The overall association between FF proximity and BMI was not significant but modified by sex (p-_heterogeneity_ <0.001). An exponential decrease in FF proximity was associated with 0.7 kg/m^2^ (p < 0.001) increase in BMI among males and 0.4 kg/m^2^ (p < 0.05) decrease among females. Compared to those with no FF access, males with Low, Moderate, and High FF access had higher BMI (0.6, 0.6, and 1.0 kg/m^2^, p_-trend _= 0.001), while females had lower BMI (−0.3, −0.6, and −0.9 kg/m^2^, p_-trend _< 0.001). The findings suggest that environmental exposures do not affect all population groups equally. Therefore, public health strategies and future research on obesogenic environments should consider the influence of social, cultural, and the broader environmental factors.

## Introduction

The increasing prevalence of obesity had coincided with the proliferation of fast-food restaurants in low- and middle-income countries. The expansion of the Western fast-food industry due to globalization were in parallel with the nutrition transition in these countries [1,2]. Moreover, urbanization also had resulted in a trend of eating out, as the increased density of food outlets and time constraints imposed by longer working hours have created a culture of convenience [3]. Healthy lifestyle choices can attenuate obesity but are often challenged by an environment that enables and reinforces preferences for unhealthy foods [4]. It is imperative to recognize the pivotal role of the food environment in the development of obesity.

Fast-food is commonly known as calorie-dense food produced in bulk by major franchises using cheap ingredients, flavour enhancers, preservatives, and additives to enhance palatability and satiety [5]. Frequent and long-term consumption of fast-food such as processed red meat, fries, burgers, fried chicken, and fried foods increased the risk of obesity [6]. Moreover, fast-food increases the preference for fried and sweet foods, besides lowering the overall diet quality [7]. Over the years, fast-food restaurants have increased their variety, portion size, energy, and sodium content while having limited availability of healthy choices [8]. Consequently, access to fast-food restaurants has been commonly documented as an obesogenic environment factor [9]. The probability of obesity among the UK population tripled when the genetic predisposition to obesity was exacerbated by substantial exposure to fast-food establishments [10]. In China, the proliferation of Western fast-food establishments has counteracted the favourable health consequences associated with walkable urban spaces and convenient access to traditional produce and vegetable markets [11]. The evidence linking the accessibility of fast-food and obesity generally remains inconsistent and varies across the geography and socio-demographics of the study population [12]. Personal, cultural, and environmental factors interact to shape dietary behaviour, which vary significantly across different populations. The effect of other food vendors and the local food environment can also influence the impact of fast-food environment in shaping dietary behaviours.

Nationwide surveys in Malaysia have reported a tremendous increase in the prevalence of overweight and obesity in the past three decades from 21% in 1996 to 54.4% in 2023 [13]. An estimated 40% of Malaysians consume meals outside their homes daily, accounting for 21% of their household expenditure, while 95% of Malaysian adults reported inadequate fruit and vegetable intake [14]. Compared to nearby countries, Malaysia experienced a greater increase in the number of KFC, McDonald’s, and Pizza Hut establishments per capita between 2004 and 2017 [15]. Among adults, the prevalence of fast-food consumption at least once a week was 17.4% and 25% among suburban and urban areas, respectively [16,17]. However, evidence of environmental influence as a predisposition to obesity in Malaysia is limited. Local studies have concentrated on the effect of fast-food environments on obesity among children and adolescents [18,19]. There have been no studies on its impact on adults, who have a more significant influence over the food preferences of their families, peers, and communities because of their higher financial capacity. This study aimed to assess the association between access to fast-food restaurants and body mass index among adults aged 18–59 years in Malaysia nationwide.

## Materials & methods

### Study population

This study utilized data from the National Health and Morbidity Survey (NHMS) 2015, a cross-sectional survey of nationally representative community-dwelling non-institutionalized adults in Malaysia. Although more recent iterations of the National Health and Morbidity Survey (NHMS), have been conducted, the NHMS 2015 dataset was utilized for this study due to its relevance, completeness, and accessibility at the time of analysis. Despite being conducted a decade ago, the data remain highly valuable for understanding long-term health patterns and informing public health policy. The 2015 survey also employed standardized methodologies aligned with previous and present national surveys, allowing for robust comparisons and consistent trend analysis. Furthermore, at the time of analysis, NHMS 2015 was the most recent dataset with full public access and complete documentation, making it the most suitable choice to ensure the reliability and validity of our findings.

Out of the initial 19,935 respondents in the NHMS 2015 dataset, only a total of 14,584 individuals were included in the final analysis. The current study excluded 3790 respondents aged 60 years and above to focus on the non-elderly population and ensure consistency in dietary and anthropometric patterns. Subsequently, 1007 respondents with missing weight or height data were excluded as BMI could not be calculated. Additionally, 554 respondents residing on Langkawi, Labuan and Penang islands were excluded to facilitate a standardized estimation of food environment beyond administrative boundaries. These exclusions were necessary to maintain the validity and comparability of the analyses and to ensure that the study population aligned with the research objectives.

The details of the survey were described in the publicly available official technical report ( [20]). In brief, the NHMS applied a stratified two-stage cluster sampling design, covering all 13 states and 3 federal territories in Malaysia, with stratification by urban and rural areas. The enumeration blocks (EBs), which were areas selected based on national population census, were randomly selected in the first stage, followed by living quarters (LQs) in the second stage. Structured questionnaires on socio-demography were used during face-to-face interviews, and anthropometry was measured using the Tanita Personal Scale HD 319 for weight and the SECA Stadiometer 213 for height. GPS coordinates were either detected using the data collection device or a Garmin GPS handheld device at rural areas with no Internet connection or street networks.

### Environmental characteristics

The location urbanity of respondents was pre-determined at the sampling stage. Urban was defined as a delineated area with more than 10,000 populations, and less than 60% was involved in agricultural activities [21]. Urban area referred to towns, cities and suburbs with more structures such as houses, commercial buildings, roads, bridges and railways. The district population density and district median household income variables were extracted from the Population and Housing Census 2010 and the Household Income Survey 2014, respectively, from the official website of the Department of Statistics Malaysia. The district population density was categorized as less than 150, 150–500, and more than 500 persons per km^2^, based on the classification of local councils as city, municipal, or rural districts. The district median household income represents the district’s socioeconomic status. District median household income was categorized into four groups (low, lower-middle, upper-middle, and high) using the natural Jenks method in the ArcMAP software, in which similar values were grouped.

Eight popular fast-food franchises in Malaysia, namely Kentucky Fried Chicken (KFC), McDonald’s, A&W, Burger King, Pizza Hut, Domino’s Pizza, Marrybrown, and SugarBun were selected for this study based on their extensive nationwide presence and longstanding impact in the country. The addresses of the fast-food outlets were sourced from the official websites of each franchise, verified using Google Earth, and manually geocoded using Google Maps. The operational dates of the outlets were obtained through franchise announcements on social media. To ensure the outlets were active during the study period, any outlet identified as having commenced operations after this timeframe was excluded from the analysis. In cases where operational dates could not be determined, it was assumed that the outlet was operational during the study period.

### Ethics approval of research

The present study protocol was reviewed and approved by the University of Malaya Research Ethics Committee (UM.TNC 2/UMREC-229) on July 23, 2017, and registered in the National Medical Research Register (NMRR-17-2111-36007). The data for this study was obtained from the National Institute of Health data repository on August 7, 2017. To ensure confidentiality, all individual respondents were de-identified. Only the locations and socio-demographic information were made available.

The National Health and Morbidity Survey (NHMS) 2015 was conducted by the Ministry of Health and approved by the Medical Research and Ethics Committee of the Ministry of Health Malaysia. Consent was obtained during the data collection. An information sheet and consent form were provided to all respondents. For illiterate respondents, consent was obtained through a thumbprint, witnessed by a literate individual. Before providing consent, respondents were informed that participation was voluntary and withdrawal was possible at any time without affecting their medical or health benefits. Further details of the consent and data collection process can be found in the NHMS 2015 technical report [20]. The current study was conducted as a secondary analysis and did not receive any financial support.

### Data management

The sociodemographic characteristics in terms of sex, age, ethnicity (Malay, Chinese, Indian, Indigenous groups, and others), and marital status (married or non-married) were based on the classifications used in the primary study. The highest education levels obtained were categorized as tertiary education, completed secondary education (12 years of formal education), and others (<12 years, unknown or no formal education). The body mass index (BMI) was calculated as weight (kg)/ height (m)^2^ for each respondent.

The location coordinates of fast-food restaurants and respondent’s residence were transformed into the Kertau RSO Malaya (Meters) coordinate system from its original WGS 1984 geographic system. The latitude and longitude of respondent’s residence were verified against their respective enumeration block (EB) to ensure accuracy. Wrong or missing coordinates were imputed using the coordinates of respondents from the same EB. The coordinates were truncated to three decimal points, equivalent to 100 meters on land for privacy protection.

Fast-food proximity was calculated as the Euclidian distance to the nearest fast-food restaurant from each respondent’s home location using the ‘Proximity’ function. Fast-food proximity was also categorized as ≤1 km, 1–3 km, and >3 km to represent walking, nearby, and further distances, respectively. Previous research has established a standard of 1 km, equivalent to 10 minutes of brisk walking, and 3 km, which is a distance that can be easily travelled by car [22,23]. Additionally, prior local studies have utilized 1 km as a benchmark for measuring access to fast-food, enhancing the comparability of this study [18,19]. Furthermore, a 3-km buffer distance was recommended for restaurants and 8-km for stores, as 8 km was noted as the furthest distance that people would travel to procure food [24].

**Fig 1:**
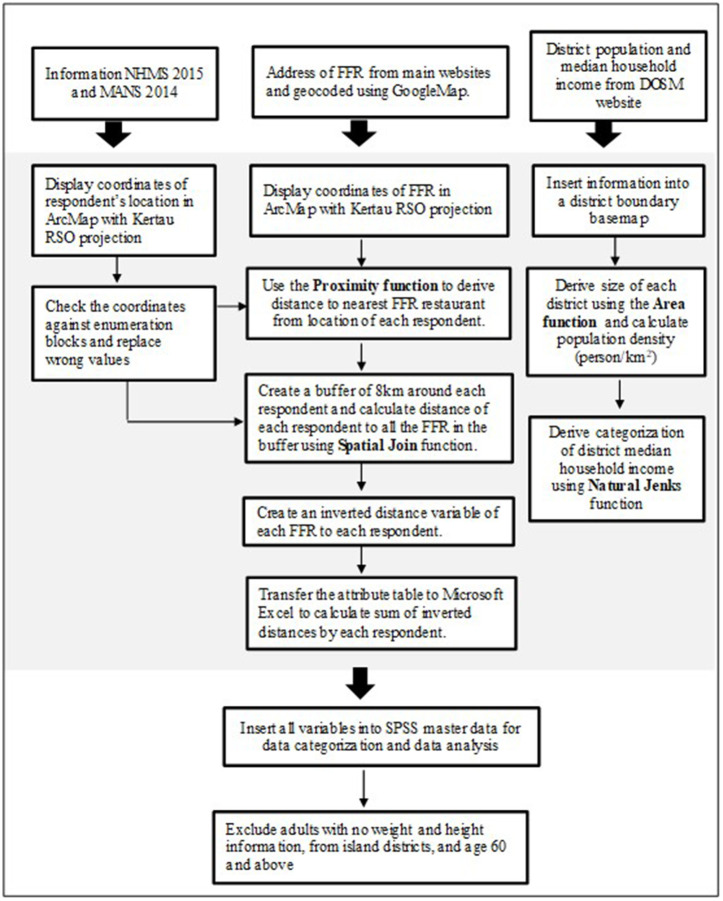
Data management process workflow. * DOSM = Department of Statistics Malaysia (https://newss.statistics.gov.my/).

Subsequently, an 8-km buffer distance for each respondent was established as the possible area of influence for the spatial access to food. The distance-decay principle, which states diminishing access with increasing distance, was used to estimate spatial access. The distances to all fast-food restaurants within the 8-km buffer were measured. Each fast-food restaurant was subsequently weighted according to an inverse distance function (1/distance). The inverse distances to each fast-food restaurant were then summed and allocated to each respondent. The scores were categorized to better interpret their meanings. Respondents with a score of zero were categorized as none indicating no access to fast-food restaurants within 8-km distance. The remaining scores were further categorized into low, moderate, and high spatial access based on tertile cut-offs [25,26]. All spatial analysis used to derive fast-food accessibility measures (proximity and spatial access), district population density, and district median household income was conducted using the ArcMap 10.7 software. Fig 1 illustrates the data management process.

### Statistical analysis

Descriptive statistics were summarized using proportions for categorical data , and group differences were evaluated using the chi-square test. Continuous variables were summarized as means and standard deviations (SD), and their differences were tested with t-test. FF proximity with right-skewed distributions were summarized as medians and interquartile ranges, and differences between the medians were tested using the Kruskal-Wallis test.

Mixed-effect linear regression was applied to account for the unexplained variability in the clustering of respondents at the enumeration block level. The analysis assumed that coefficients at the individual level were fixed, whilst coefficients at the clustering level were random, resulting from the factors that were not included in the explanatory variables used. A mixed model regression provided a more accurate estimate by correcting for underestimated standard errors which could lead to overstatement of statistical significance. In this study, a linear multilevel model with robust standard error estimation was applied to account for the hierarchical structure of the data, where individuals were nested within enumeration blocks (EBs) (n = 834). Maximum likelihood with robust estimation was applied to handle violations of model assumptions. Robust standard errors were recommended in cases of unequal groups at Level 2 and in the presence of a low intraclass correlation (ICC), which may lead to biased estimates of the second-level standard errors, affecting the normality assumption of the level-2 error distribution. The intraclass correlation (ICC) value was calculated as the ratio of the between-cluster variance to the total variance (within and between variance) obtained by fitting an empty model. In this study, the intraclass correlation coefficient (ICC) derived from the empty (null) model was 0.045, indicating that 4.5% of the total variance in BMI was attributable to differences between EBs. In practice, ICC was usually low, but clustering must still be accounted, to quantify for the “contextual phenomena” where people from the same neighbourhood were more like each other in terms of health outcome than to people from different neighbourhoods. 

Fast-food environment as the exposure variable was represented using three operational measures: [1] continuous log-transformed fast-food proximity, [2] categorical fast-food proximity, and [3] categorical fast-food spatial access. Guided by the socio-ecological framework, the fast-food environment was conceptualized as a distal factor in relation to BMI, potentially confounded by individual and area-level socio-demographic characteristics. Therefore, to adjust for potential confounders, two sequential models associating fast-food environment and BMI were constructed:

Model 1: Adjusted for individual-level characteristics, including age, sex, ethnicity, education level, employment status, household income level, and marital status.

Model 2: Further adjusted for area-level characteristics, including Peninsular or East Malaysia region, urban/rural classification, district population density, and district household median income, to account for contextual socio-economic differences.

The model performance for all three exposure variables was assessed using changes in Akaike Information Criterion (AIC) and Bayesian Information Criterion (BIC). Model 1 consistently had lower AIC and BIC values across all exposure types, suggesting a better statistical fit and greater parsimony due to fewer parameters. However, the AIC and BIC differences between Model 1 and Model 2 were modest, indicating only a minor improvement in fit. Model 2 offered a more comprehensive, multilevel ecological perspective on BMI which are essential for understanding broader structural and contextual influences on BMI that go beyond individual-level factors.

Interactions between fast-food spatial access and socio-demographic factors were indicated using test for homogeneity of variances and evaluated using linear combinations of regression coefficients. Only the interaction with sex was statistically significant (p < 0.05), and this was further explored in stratified models. Sensitivity analysis using waist circumference as an alternative outcome yielded similar results. All statistical analyses were performed using STATA version 16.

## Results

### Characteristics of study population

The total number of NHMS 2015 respondents included in this study was 14,584 and 51.5% were females with a mean age of 38 years. Majority were Malay ethnicity, married, employed, attained secondary level education, and from Peninsular Malaysia. Males were younger, with a higher proportion of being unmarried, employed and had lower BMI than females. The proportion of males and females was similar across Peninsular and East Malaysia region, urbanity, and district median household income categories. The median fast-food proximity of all respondents was 2.6 km, and was similar between males and females (Table 1).

**Table 1:** Characteristics of respondents by male and female.

Characteristics	All (N = 14584)	Male (n = 7080)	Female (n = 7504)
Age, years; Mean (SD)	38.0 (12.0)	37.4 (12.1)	38.5 (11.9) ***
Ethnicity			
Malay	9060 (62.1)	4365 (61.7)	4695 (62.6) ***
Chinese	1961 (13.4)	973 (13.7)	988 (13.2)
Indian	1063 (7.3)	485 (6.9)	578 (7.7)
Indigenous	1396 (9.6)	640 (9.0)	756 (10.1)
Others	1104 (7.6)	617 (8.7)	487 (6.5)
Marital Status			
Not married	4485 (30.8)	2299 (32.5)	2186 (29.1) ***
Married	10099 (69.2)	4781 (67.5)	5318 (70.9)
Occupation			
Government	1986 (13.6)	1,011 (14.3)	975 (13.0) ***
Private	5290 (36.3)	3256 (46.0)	2034 (27.1)
Self-employed	2991 (20.5)	1933 (27.3)	1058 (14.1)
Unemployed	2,668 (18.3)	216 (3.1)	2452 (32.7)
Education Level			
Tertiary	3687 (25.3)	1725 (24.4)	1962 (26.1) ***
Secondary	7526 (51.6)	3785 (53.5)	3741 (49.9)
Others	3371 (23.1)	1570 (22.2)	1801 (24.0)
Household income, RM			
Median (IQR)	3000 (3800)	3300 (3840)	3000 (3750) ***
Region			
Peninsular Malaysia	12337 (84.6)	6026 (85.1)	6311 (84.1)
East Malaysia	2247 (15.4)	1054 (14.9)	1193 (15.9)
Urbanity			
Rural	5892 (40.4)	2851 (40.3)	3041 (40.5)
Urban	8692 (59.6)	4229 (59.7)	4463 (59.5)
District population density			
Least	4933 (33.8)	2425 (34.3)	2508 (33.4) **
Moderate	3893 (26.7)	1788 (25.3)	2105 (28.1)
Most	5758 (39.5)	2867 (40.5)	2891 (38.5)
District median HH income			
Low	1423 (9.8)	662 (9.4)	761 (10.1)
Lower middle	5676 (38.9)	2748 (38.8)	2928 (39.0)
Upper middle	4609 (31.6)	2223 (31.4)	2386 (31.8)
High	2876 (19.7)	1447 (20.4)	1429 (19.0)
Fast-food proximity; km Median (IQR)	2.6 (6.4)	2.6 (6.4)	2.6 (6.4)
BMI, kg/m^2^; Mean (SD)	26.0 (5.5)	25.4 (5.1)	26.5 (5.9) ***

^a^ All values are expressed as n (%) unless stated otherwise.

** p < 0.01; *** p < 0.001 indicated significant mean differences between male and female

All individual and area socio-demographic characteristics, except sex, differed between the categories of fast-food proximity and fast-food access. Closer fast-food proximity and higher fast-food spatial access was found among younger, tertiary-educated, higher household income, and government or private employment respondents. Areas closer to fast-food restaurants had higher proportion of Chinese and Indian ethnicity. Majority of respondents with high fast-food spatial access and close fast-food proximity were at Peninsular Malaysia, urban areas, district with most population density. There was also a trend of increasing fast-food environment with district median income. The BMI of all respondents were not statistically different across the fast-food proximity and fast-food spatial access categories (Table 2).

**Table 2:** Fast-food proximity and spatial access by characteristics of respondents (N = 14,584).

Characteristics	Fast-food proximity			Fast-food spatial access			
	**≤ 1 km**	**1-3 km**	**> 3 km**	**None**	**Low**	**Moderate**	**High**
All	3461 (23.7)	4410 (30.2)	6713 (46.0)	3316 (22.7)	3712 (25.5)	3798 (26.0)	3758 (25.8) ^#^
Sex							
Male	1682 (48.6)	2150 (48.8)	3248 (48.4)	1609 (48.5)	1803 (48.6)	1837 (48.4)	1831 (48.7)
Female	1779 (51.4)	2260 (51.2)	3465 (51.6)	1707 (51.5)	1909 (51.4)	1961 (51.6)	1927 (51.3)
Age (years), Mean (SD)	37.0 (11.7)	37.9 (11.9)	38.5 (12.3) #	38.8 (12.3)	38.3 (12.4)	38.1 (11.8)	36.8 (11.5) #
Ethnicity							
Malay	1721 (49.7)	2785 (63.2)	4554 (67.8) #	2172 (65.5)	2664 (71.8)	2303 (60.6)	1921 (51.1) ^#^
Chinese	862 (24.9)	701 (15.9)	398 (5.9)	142 (4.3)	283 (7.6)	625 (16.5)	911 (24.2)
Indian	366 (10.6)	378 (8.6)	319 (4.8)	59 (1.8)	313 (8.4)	302 (8.0)	389 (10.4)
Indigenous groups	153 (4.4)	282 (6.4)	961 (14.3)	671 (20.2)	249 (6.7)	321 (8.5)	155 (4.1)
Others	359 (10.4)	264 (6.0)	481 (7.2)	272 (8.2)	203 (5.5)	247 (6.5)	382 (10.2)
Marital status							
Married	2287 (66.1)	3065 (69.5)	4747 (70.7)	980 (29.6)	1136 (30.6)	1098 (28.9)	1271 (33.8)
Non-married	1174 (33.9)	1345 (30.5)	1966 (29.3)	2336 (70.4)	2576 (69.4)	2700 (71.1)	2487 (66.2)
Education Level							
Tertiary	1143 (33.0)	1374 (31.2)	1170 (17.4) #	483 (14.6)	700 (18.9)	1037 (27.3)	1467 (39.0) ^#^
Secondary	1685 (48.7)	2245 (50.9)	3596 (53.6)	1678 (50.6)	2126 (57.3)	2027 (53.4)	1695 (45.1)
Others	633 (18.3)	791 (17.9)	1947 (29.0)	1155 (34.8)	886 (23.9)	734 (19.3)	596 (15.9)
Occupation							
Government	472 (13.6)	743 (16.8)	771 (11.5) #	343 (10.3)	468 (12.6)	564 (14.8)	611 (16.3) #
Private	1601 (46.3)	1668 (37.8)	2021 (30.1)	875 (26.4)	1163 (31.3)	1453 (38.3)	1799 (47.9)
Self-employed	559 (16.2)	742 (16.8)	1690 (25.2)	965 (29.1)	818 (22.0)	685 (18.0)	523 (13.9)
Unemployed	465 (13.4)	794 (18.0)	1409 (21.0)	720 (21.7)	783 (21.1)	710 (18.7)	455 (12.1)
Household Income, Median (IQR)	4182 (4678)	3740 (4300)	2460 (2728) #	2250 (2400)	2600 (3000)	3300 (3670)	4700 (5300) #
BMI	25.8 (5.5)	26.0 (5.5)	26.0 (5.6)	25.9 (5.7)	26.2 (5.6)	25.9 (5.5)	25.8 (5.4)
**Characteristics**	**Fast-food proximity**			**Fast-food spatial access**			
	**≤ 1 km**	**1-3 km**	**> 3 km**	**None**	**Low**	**Moderate**	**High**
Region							
Peninsular	3114 (90.0)	3854 (87.4)	5369 (80.0) ^#^	2414 (72.8)	3291 (88.7)	3184 (83.8)	3448 (91.8) ^#^
East Malaysia	347 (10.0)	556 (12.6)	1344 (20.0)	902 (27.2)	421 (11.3)	614 (16.2)	310 (8.2)
Locality							
Rural	87 (2.5)	692 (15.7)	5113 (76.2) ^#^	3086 (93.1)	2211 (59.6)	579 (15.2)	16 (0.4) ^#^
Urban	3374 (97.5)	3718 (84.3)	1600 (23.8)	230 (6.9)	1501 (40.4)	3219 (84.8)	3742 (99.6)
District population density							
Least (<150)	524 (15.1)	971 (22.0)	3438 (51.2) ^#^	2506 (75.6)	1424 (38.4)	834 (22.0)	169 (4.5) ^#^
Moderate (150–500)	580 (16.8)	1133 (25.7)	2180 (32.5)	657 (19.8)	1613 (43.5)	1317 (34.7)	306 (8.1)
Most (>500)	2357 (68.1)	2306 (52.3)	1095 (16.3)	153 (4.6)	675 (18.2)	1647 (43.4)	3283 (87.4)
District median income							
Low	115 (3.3)	214 (4.9)	1094 (16.3) ^#^	758 (22.9)	535 (14.4)	130 (3.4)	0 ^#^
Lower middle	614 (17.7)	1583 (35.9)	3479 (51.8)	1839 (55.5)	1835 (49.4)	1812 (47.7)	190 (5.1)
Upper middle	1042 (30.1)	1481 (33.6)	2086 (31.1)	719 (21.7)	1258 (33.9)	1534 (40.4)	1098 (29.2)
High	1690 (48.8)	1132 (25.7)	54 (0.8)	0	84 (2.3)	322 (8.5)	2470 (65.7)

# p < 0.001 based on Chi-square test or Kruskal–Wallis test;

Values expressed as % column, unless specified otherwise.

### Association between fast-food environment and body mass index

There was no significant association between the three fast-food environment measures and BMI among all respondents. Model 1 for log-transformed FF proximity showed lower AIC and BIC values (AIC = 90245, BIC = 90405) compared to Model 2 (AIC = 90249, BIC = 90462), but the differences were modest, suggesting marginal improvement in model fit. Similar patterns were observed for categorical FF proximity (Model 1: AIC = 90250, BIC = 90417; Model 2: AIC = 90252, BIC = 90472) and FF spatial access (Model 1: AIC = 90249, BIC = 90424; Model 2: AIC = 90254, BIC = 90481).

However, the association between fast-food environment and BMI was significantly modified by sex (p-heterogeneity <0.001). Every log increase in fast-food proximity was associated with 0.7 kg/m^2^ decrease in BMI among males and 0.4 kg/m^2^ increase in BMI among females after adjusting for individual and area characteristics. Compared to those who were >3 km further from nearest fast-food restaurant, males and females at ≤ 1 km and 1–3 km had higher and lower BMI, respectively. Males with high fast-food spatial access within the 8 km vicinity had 1.0 kg/m^2^ higher BMI, while females had 0.9 kg/m^2^ lower BMI compared to their counterparts with no fast-food spatial access, after adjusting for individual and area characteristics. The dose-response trend for fast-food proximity and fast-food spatial access was significant for males and females (Table 3).

**Table 3:** Association between fast-food accessibility and body mass index.

	All ^c^	Male	Female	P_heterogeneity_
	Δ BMI, kg/m^2^, Mean ± SE (95% CI)			
**FF Proximity (log)**				
Crude Model	−0.1 (−0.2, 0.2)	−0.6 (−0.8, −0.3)	0.6 (0.3, 0.9)	***
Model 1^a^	−0.2 (−0.4, 0.1)	−0.7 (−1.0, −0.5) ***	0.3 (0.1, 0.6) **	***
Model 2^b^	−0.2 (−0.5, 0.1)	−0.7 (−1.0, −0.4) ***	0.4 (0.1, 0.7) *	***
**FF Proximity**				
Crude Model				
≤ 1 km	−0.3 (−0.6, 0.1)	0.4 (0.1, 0.8) *	−0.9 (−1.3, −0.6) ***	***
1- 3 km	−0.1 (−0.4, 0.2)	0.4 (0.1, 0.8) *	−0.6 (−0.9, −0.2) **	
> 3 km	0 (ref.)	0 (ref.)	0 (ref.)	
Model 1^a^				
≤ 1 km	0.1 (−0.2, 0.4)	0.7 (0.3, 1.1) ***	−0.5 (−0.9, −0.2) **	***
1- 3 km	−0.1 (−0.3, 0.2)	0.5 (0.2, 0.8) **	−0.5 (−0.8, −0.1) **	
> 3 km	0 (ref.)	0 (ref.)	0 (ref.)	
Model 2 ^b^				
≤ 1 km	−0.1 (−0.5, 0.3)	0.5 (0.1, 1.0) * ^d^	−0.7 (−1.1, −0.3) ** ^d^	***
1- 3 km	−0.1 (−0.4, 0.2)	0.4 (0.1, 0.7) *	−0.6 (−1.0, −0.2) **	
> 3 km	0 (ref.)	0 (ref.)	0 (ref.)	
**FF Spatial access**				
Crude Model				
None	0 (ref.)	0 (ref.)	0 (ref.)	***
Low	0.3 (−0.1, 0.6)	0.7 (0.3, 1.2) **	−0.1 (−0.6, 0.3)	
Moderate	0.1 (−0.3, 0.4)	0.7 (0.3, 1.2) **	−0.6 (−1.1, −0.2) **	
High	−0.1 (−0.4, 0.3)	1.0 (0.5, 1.4) ***	−1.0 (−1.5, −0.6) ***	
Model 1^a^				
None	0 (ref.)	0 (ref.)	0 (ref.)	***
Low	0.2 (−0.1, 0.5)	0.6 (0.2, 1.1) **	−0.2 (−0.6, 0.2)	
Moderate	0.1 (−0.3, 0.4)	0.7 (0.3, 1.1) ***	−0.5 (−1.0, −0.1) *	
High	0.3 (−0.1, 0.6)	1.2 (0.8, 1.6) ***	−0.7 (−1.2, −0.3) **	
Model 2 ^b,^				
None	0 (ref.)	0 (ref.) ^e^	0 (ref.) ^e^	***
Low	0.1 (−0.2, 0.5)	0.6 (0.1, 1.1) *	−0.3 (−0.7, 0.2)	
Moderate	−0.1 (−0.5, 0.4)	0.6 (0.1, 1.1) *	−0.6 (−1.2, −0.1) *	
High	0.1 (−0.5, 0.6)	1.0 (0.4, 1.6) **	−0.9 (−1.5, −0.3) **	

^a^ Model 1: Adjusted for individual-level characteristics, including age, ethnicity, education level, employment status, household income level, and marital status.

^b^ Model 2: Adjusted for individual-level and area-level characteristics, including Peninsular or East Malaysia region, urban/rural classification, district population density, and district household median income.

^c^ Sex was adjusted in the analysis for all respondents.

*’ p < 0.05; ** p < 0.01; ***p < 0.001

^d^ dose-response trend for FF Proximity (Model 2) was significant for males (p = 0.01) and females (p < 0.001).

^e^ dose response trend for FF Spatial access (Model 2) was significant for males (p = 0.001) and females (p < 0.001).

**Fig 2:**
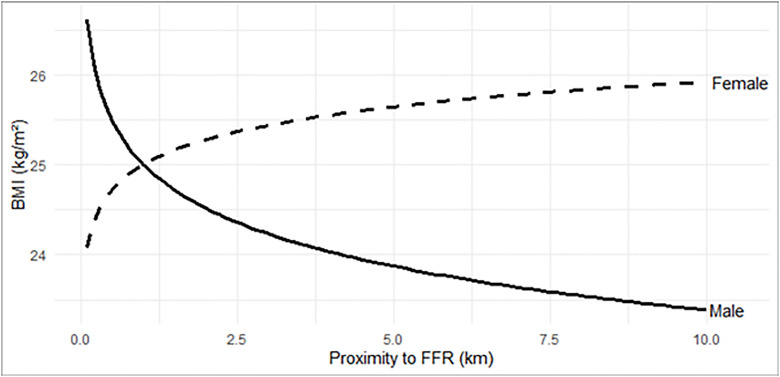
Association between FF Proximity and BMI by sex.

Fig 2 illustrates the contrasting trend between male and female in the association between FF proximity and BMI. With one log unit closer to fast-food proximity, males had 0.7 kg/m^2^ higher BMI. Conversely, females had 0.4 kg/m^2^ lower BMI, independent of individual socio-demography, district population density, and district median household income (Fig 2).

**Fig 3:**
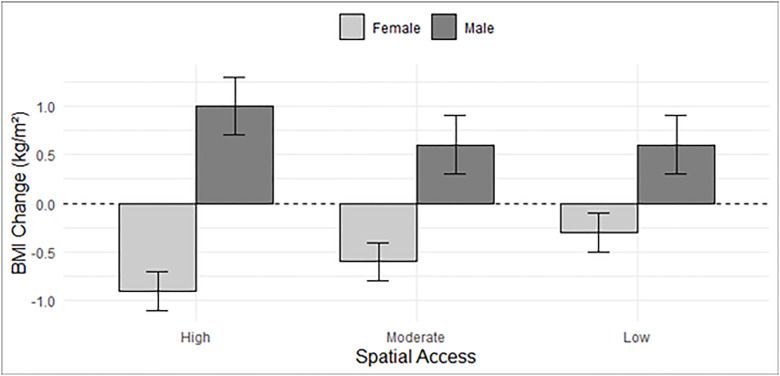
Association between FF spatial access and BMI by sex.

Fig 3 illustrates the BMI comparison between male and female and different FF spatial access. Males at low, moderate, and high fast-food spatial access also had significantly higher BMI than those with none access respectively. In contrast, females reported a decreasing BMI trend from None, Low, Moderate to High spatial access (Fig 3).

## Discussion

The present study demonstrated a contrasting relationship of the fast-food environment with BMI in males and females. Sex-specific in the relationship with food environment have been reported in other studies. An intervention study in the United States revealed that their weight loss program was less effective for males living near fast-food restaurants but not for females [27]. National survey in China have reported a fast-growing waistline among relatively lean males in mid-income households [28]. In a separate study, An and colleagues have found that higher access to fast-food environment was linked to increased obesity in males but decreased obesity in females, particularly in urban areas [12]. The prevalence of obesity among middle-aged males in China have been positively linked to unhealthy food environment, while obesity among females was negatively associated with the density of fresh food markets [29]. The link between a dense presence of fast-food outlets and increased weight were more pronounced among individuals with both high access to fast-food restaurants and high fast-food consumption [30,31]. Males generally favour fast-food because of its convenience and enhanced flavour, often dismissing its health implications [32]. Males also were reported to be more responsive to stimuli from fast-food restaurants [33]. Inconsequentially, the global increase in obesity-related deaths and burden of disease were evidently greater among males [34].

In Malaysia, fast-food restaurants are typically located at convenient locations or equipped with drive-thru services and offering meals at affordable prices with special promotions during selected hours. A higher proportion of males compared to females in the present study were employed. Working adults tended to eat out frequently, even after adjusting for education, income, and demographics [35]. Ashari and colleagues found that males were more inclined to dine out with their peers [36]. Working males may be more likely to consume fast-food when it’s easily available, due to its convenience. Moreover, compared to females who were more likely to consume fruits, vegetables, milk, milk products, and cereals, Malaysian males have reported higher intake of energy and consume more carbohydrate-based foods, meat, fish, fats, oils, and sweets [37]. Females being more selective in their food choices, could possibly be because they are more likely to succumb to social and cultural pressures to maintain body weight [38]. This suggests that, given the wide array of food choices typically available in urban areas, males would be more likely to opt for convenient and satiating foods.

The present study found that every one-fold closer in fast-food proximity was associated with 0.7 kg/m^2^ increase in BMI and fast-food proximity within 1 km was associated with a 0.5 kg/m^2^ higher BMI compared to further than 3 km. The magnitude of BMI change found among males in this study was comparable to studies associating fast-food intake or its accessibility with BMI. A study in the United States found that an increase of one meal per week in a fast-food restaurant was associated with a 0.8 kg/m^2^ BMI increase [39]. Another study in Australia found that relative exposure to 25% more fast-food outlets was associated with a 0.4 to 1.2 kg/m^2^ increase in BMI [22]. However, a lower impact on BMI had also been reported in other populations. Among UK Biobank respondents, 0.24 kg/m^2^ higher BMI was reported for every 10-fold nearer in fast-food proximity [40]. Fast-food proximity within 1 km was associated with a 0.3 kg/m^2^ higher BMI in the urban areas of the Netherlands [41]. In South Africa, each kilometre nearer of fast-food proximity increased BMI by 0.014 kg/m^2^ before adjusting for confounders [42]. Evidently, the effect of fast-food environment on obesity is specific to study populations.

Food environment varies across populations. Fast-food restaurants may constitute a greater proportion of restaurants in areas with low to moderate economic activity. Residents in areas with a predomination of fast-food restaurants had a 2.5 times greater risk of obesity compared to those less exposed [43]. Higher access to fast-food restaurants also had greater access to similar restaurants and food stores with cheaper options of similar high-calorie foods in the same area [44]. Greater neighbourhood spatial access to restaurants was associated with a lower frequency of home cooking, independent of access to grocery stores [26]. This indicates that food environment in restaurants will affect the dietary behaviour of the population. Governments in some countries have worked with the fast-food industry to regulate the reformulation of nutrient content by introducing options with less fat, less sugar, less sodium, and increased fibre in addition to enhancing labelling legislation [45]. A few local governments in England have regulated the establishment of new fast-food outlets if obesity levels exceeded a certain threshold or if there was already a high concentration of fast-food establishments [46]. The land-use policy have effectively reduced the number of fast-food outlets, thus limiting access to unhealthy foods in the local environment [47]. Although this method may not be applicable to all populations, but it underscores the role of local governments in creating healthier food environments.

The strength of the present study was the use of spatial access measures within a large buffer size, in addition to fast-food proximity in quantifying the fast-food environment. The application of several matrices was recommended because the appropriateness of any environmental measure depends on the specific population [48]. Varying buffer sizes were often used for different food store types and populations with different abilities to procure food, accounting for the walking time [22]. Defining neighbourhood of influence could be difficult as individuals often move outside a radial buffer or an administratively defined area [31]. Adults aged 18–60 years were typical for car ownership, and the 8 km Euclidean buffer was considered the maximum distance the study population was likely to travel to procure food [24]. Therefore, spatial access measures were practical, accounting for both the distance from the participant’s home address to each fast-food restaurant and the total number of fast-food restaurants in the individual’s neighbourhood. Furthermore, the 1 km and 3 km thresholds used are appropriate for the Malaysian context, as the study encompasses both urban and rural settings nationwide. Fast-food restaurants are typically located in town centres, neighbourhood hubs, petrol stations, or shopping malls. Living within 1 km likely reflects frequent access, as this distance translates to a short 5-minute drive or a 10–15-minute walk. Beyond 3 km, access becomes less convenient, indicating over 30 minutes’ drive in urban areas, or an occasional visit combined with other errands in rural areas. However, the growing availability of food delivery services, particularly in urban areas, may reduce the influence of physical distance, potentially attenuating the effect of spatial proximity on dietary behaviours.

As a secondary data analysis, the present study is limited to the data available and lack of individual-level data on physical activity and underlying health status, which could influence BMI. In Malaysia, one in three adults were physically inactive, particularly among females, urban residents, older adults, individuals with higher income, married, and with long working hours [20,49]. Urban males with high fast-food access may engage in less physical activity due to sedentary jobs, limited access to recreational spaces, or long working hours, contributing to higher body weight. In contrast, rural males with less fast-food access may be more physically active due to labour-intensive occupations or greater outdoor mobility, which can help maintain a healthier weight. Furthermore, smoking was more prevalent in males in rural areas, which could partially explain the lower BMI in rural males due to its effects on appetite and metabolism [20]. For females, the reverse pattern could be possible. Urban females with higher fast-food access, may also have better access to healthier food options, gyms, parks, or structured fitness activities, which could help mitigate weight gain. Rural females may have lower physical activity levels due to household responsibilities, limited access to exercise facilities, or cultural norms that discourage outdoor activities. These factors can potentially moderate the impact of fast-food availability on body weight. Pertinently, the relationship between food environment and BMI must be interpreted within the context of other social, cultural, and behavioural factors, such as gender norms, health awareness, time constraints, income, food preferences, and physical activity levels.

Fast-food restaurants are not the only source of energy-dense and nutrient-poor foods in Malaysia. Other common food vendors, such as street hawkers, roadside stalls, and convenience stores, also sell affordable, high-fat, and high-sugar food items. Examples include fried banana fritters, *kuih lapis*, fried spring rolls, and other local snacks or cakes which are typically fried or made with coconut milk and consumed during tea breaks. These foods, often consumed with sweetened beverages, can significantly increase overall energy intake and contribute to obesity. Thus, not capturing other types of food vendors may lead to an underestimation of the true exposure to unhealthy food environments. Nevertheless, the focus on standard-classified FFR allows for comparability with previous studies conducted in other countries. popular fast-food brands FFR often proxy for unhealthy food environments, which could be due to their greater financial resilience. Furthermore, the presence of FFRs in an area often encourages the clustering of similar food outlets. Local vendors may sell comparable food items, benefiting from shared traffic [50]. This practice was common of studies in low and middle-income countries without a standard comprehensive national food retail database [42,51]. Narrow construct definitions of the retail food environment can capture a more consistent type of food provision and had produce more positive associations compared to broader definitions [31]. Despite the limited focus, the findings offer valuable insight into the potential influence of the local food environment on population-level obesity outcomes, particularly among males.

We acknowledge the lack of other environmental factors that could influence BMI, such as the presence of green spaces, blue spaces, recreation parks, public gymnasiums, walkability, and public transportation. Due to resource constraints, this study only included district population density, median household income, and location urbanity as a proxy to neighbourhood characterization. Districts with higher population density or median household income were assumed to have greater economic activity and a wider variety of stores and restaurants. Existing literature have suggested that population density and area socioeconomic status were reasonable proxies for assessing obesogenic environments. Population density have been shown to be effective predictors of walking behaviour, often outperforming more complex composite indicators [52]. Similarly, area socioeconomic status have been found to improve the explanatory power and model fit of obesogenic indices, outperforming a composite index with 17 components [53]. However, built environments may still vary across districts within the same population density and income category. Particularly, urban areas differing in terms of walkability, public transportation, road traffics, and availability of recreation parks may potentially mitigate the impact of fast-food availability by promoting higher physical activity levels. This may affect the relevance of this study’s findings in areas with extreme income levels with unusual built environments. We recognize that residual confounding and selection bias cannot be entirely ruled out by adjusting for individual- and area-level covariates. This limitation is inherent to most observational studies in this field.

The cross-sectional design of this study prevented the establishment of temporal relationships between fast-food accessibility and weight outcomes. While associations between fast-food availability and BMI were observed, these relationships could have been influenced by reverse causality. Rather than fast-food availability directly contributing to higher BMI, it is possible that areas with a higher prevalence of overweight and obese individuals attracted more fast-food outlets due to increased consumer demand. Moreover, there may be something else driving both fast-food access and obesity. Higher-income areas may have more fast-food outlets due to higher consumer spending power and commercial viability. However, individuals in these areas may also have greater access to healthier food options, recreational facilities, and healthcare, which could mitigate the effects of fast-food consumption on BMI. Conversely, in lower-income areas, limited access to nutritious food, higher stress levels, and financial constraints may contribute to obesity, regardless of fast-food availability. The interplay of these factors may obscure the true impact of the fast-food environment on BMI. Given the limitations on study designs and measurements, findings of this study should be cautiously interpreted. Nevertheless, our findings provided valuable insights into the contextual association between fast-food proximity and BMI within the Malaysian setting, and highlight the need for future work using quasi-experimental or longitudinal designs to better address causality.

## Conclusion

In conclusion, access to fast-food restaurant was associated with higher weight outcomes among adults in Malaysia where males and females demonstrated contrasting findings. The contrasting associations between fast-food proximity and BMI among males and females suggest that environmental exposures do not affect all population groups uniformly. This implies the need to develop public health strategies that are tailored to sex-specific behavioural patterns. Moreover, monitoring obesity trends by sex and geographic context can help identify at-risk groups more precisely and evaluate the impact of local interventions. Further research should seek to understand on the obesogenic effect of other environmental factors.
